# Supplementary material for: Geological evidence for extensive basin ejecta as plains terrains in the Moon’s South Polar Region
Source: Nat Commun. 2024 Jul 10;15:5783. doi: 10.1038/s41467-024-50155-w (PMC11237148; doi:10.1038/s41467-024-50155-w)
Supplement: Supplementary file 1 — Supplementary Information [file 41467_2024_50155_MOESM1_ESM.pdf]

Supplementary Information for

Geological Evidence for Extensive Basin Ejecta as Plains Terrains in the Moon's South Polar Region

Le Qiao*, Luyuan Xu, James W. Head, Jian Chen, Yuzheng Zhang, Bo Li, Zongcheng Ling

*Corresponding author. Email: leqiao@sdu.edu.cn

This PDF file includes:

Supplementary Figs. 1–15
Supplementary Table S1
Supplementary References

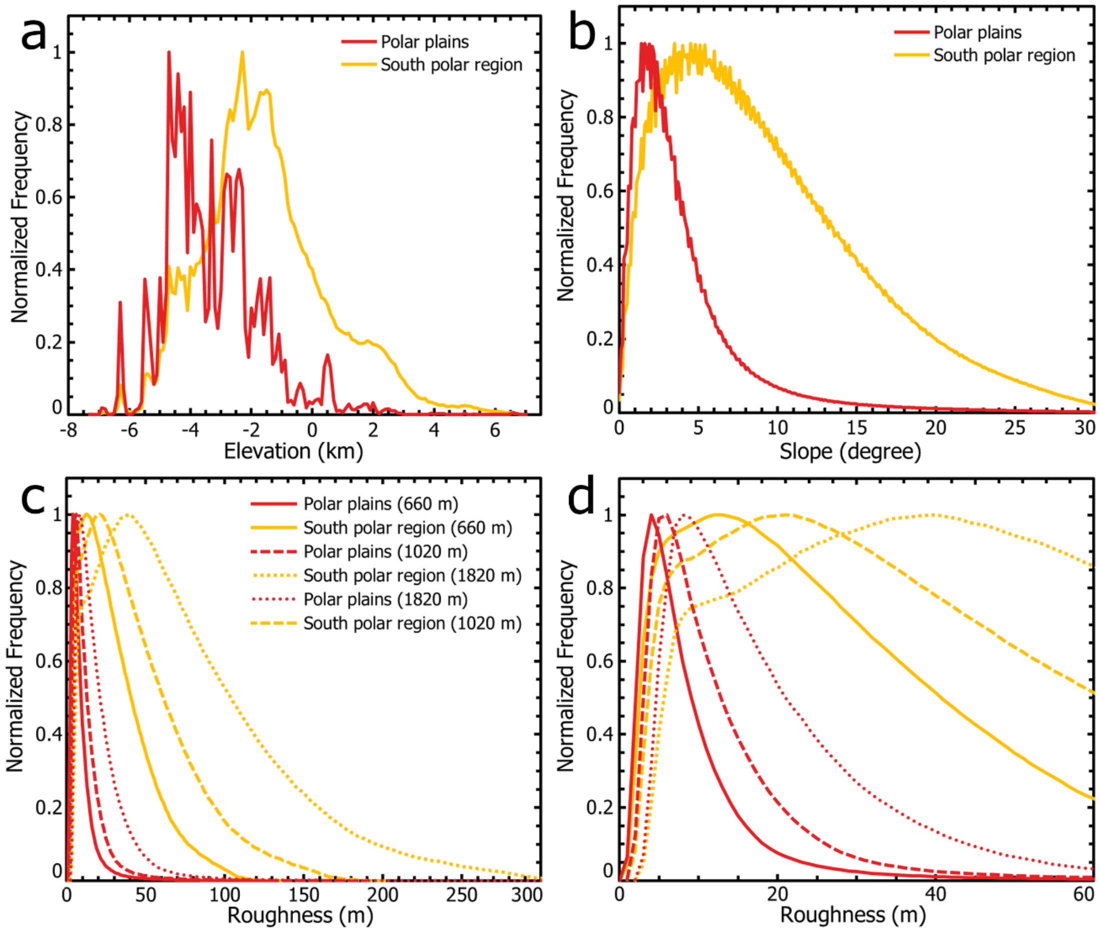

Supplementary Fig. 1. Normalized frequency of LOLA (Lunar Orbiter Laser Altimeter) (a) surface elevation, (b) slopes, and (c, d) roughness at three baselines (660 m, 1020 m, and 1820 m) for the mapped polar plains in this work and their comparison with the entire southern polar region. Panel d is a replot of panel c with smaller roughness values (horizontal axis) to highlight the roughness value difference.

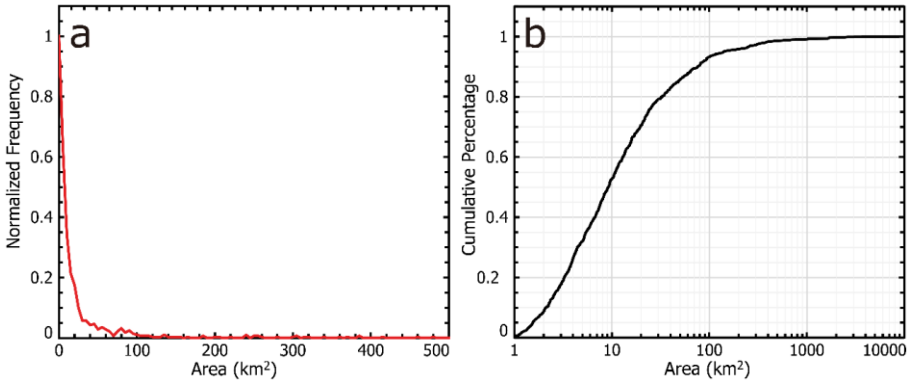

Supplementary Fig. 2. (a) Normalized frequency and (b) cumulative percentage of surface area (in km^2) of the polar plains terrains mapped in this work.

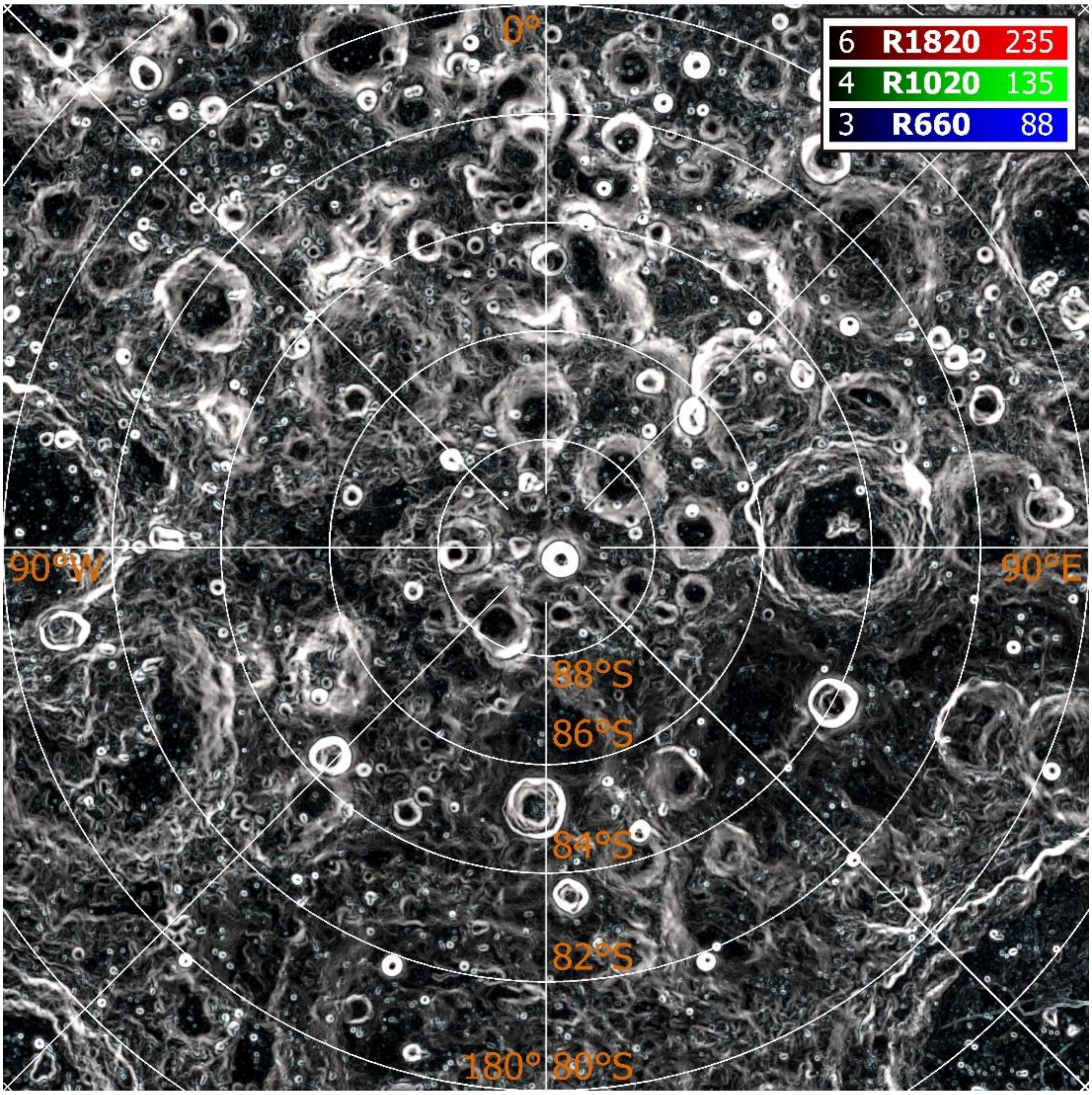

Supplementary Fig. 3. Roughness map of the southern polar region of the Moon calculated from LOLA (Lunar Orbiter Laser Altimeter) grided topography data: the red, green, and blue channels correspond to roughness (R) values (in meters) at baselines of 1820 m, 1020 m, and 660 m, respectively.

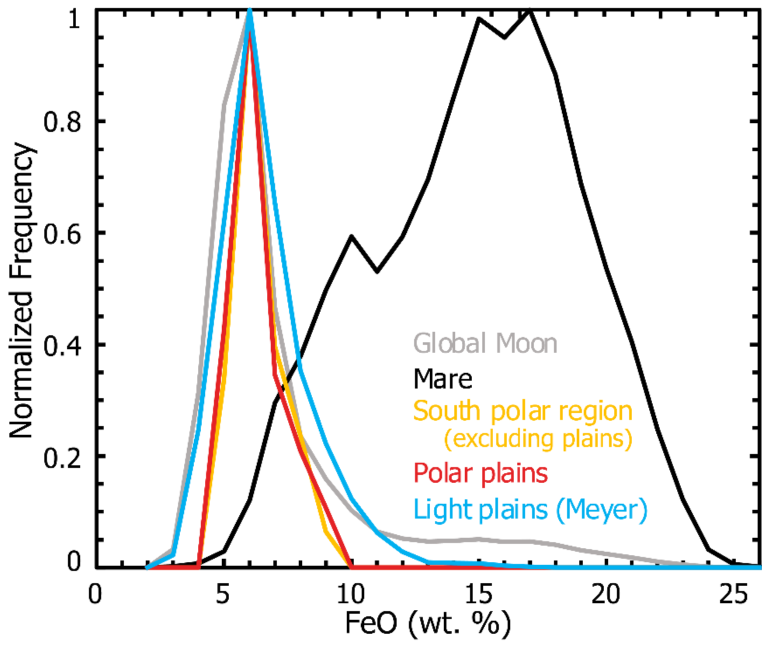

Supplementary Fig. 4. Normalized frequency of Lunar Prospector GRS (gamma ray spectrometer) FeO abundance for the polar plains mapped in this work and their comparison with those of the global Moon, lunar maria, the entire southern polar region (excluding the mapped plains), and light plains mapped by Meyer et al.[1].

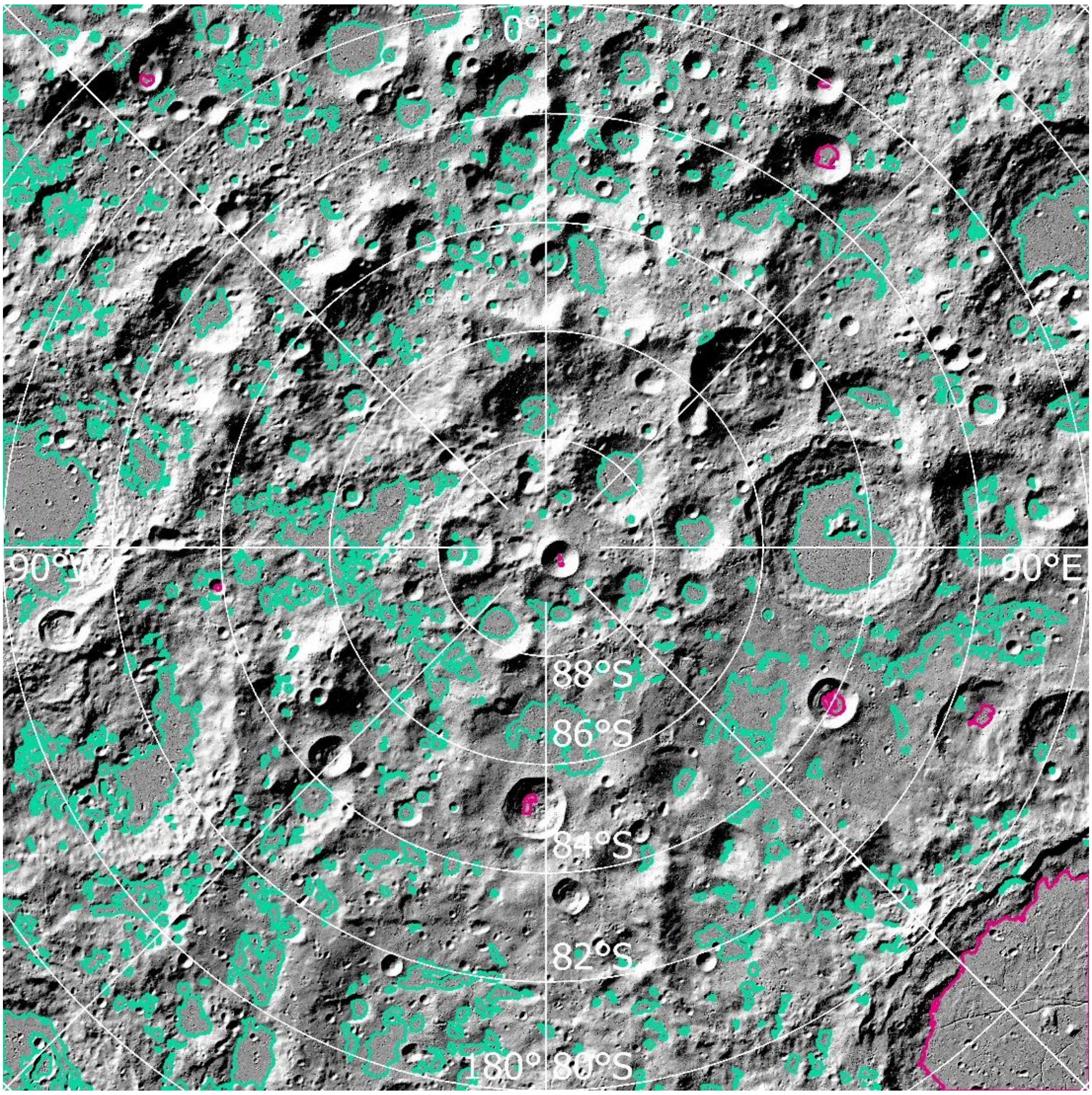

Supplementary Fig. 5. Spatial distribution of plain terrains in the southern lunar polar region in terms of the classification of their morphological characteristics: green for Amundsen-type smooth plains and pink for Schrödinger-type mounded plains.

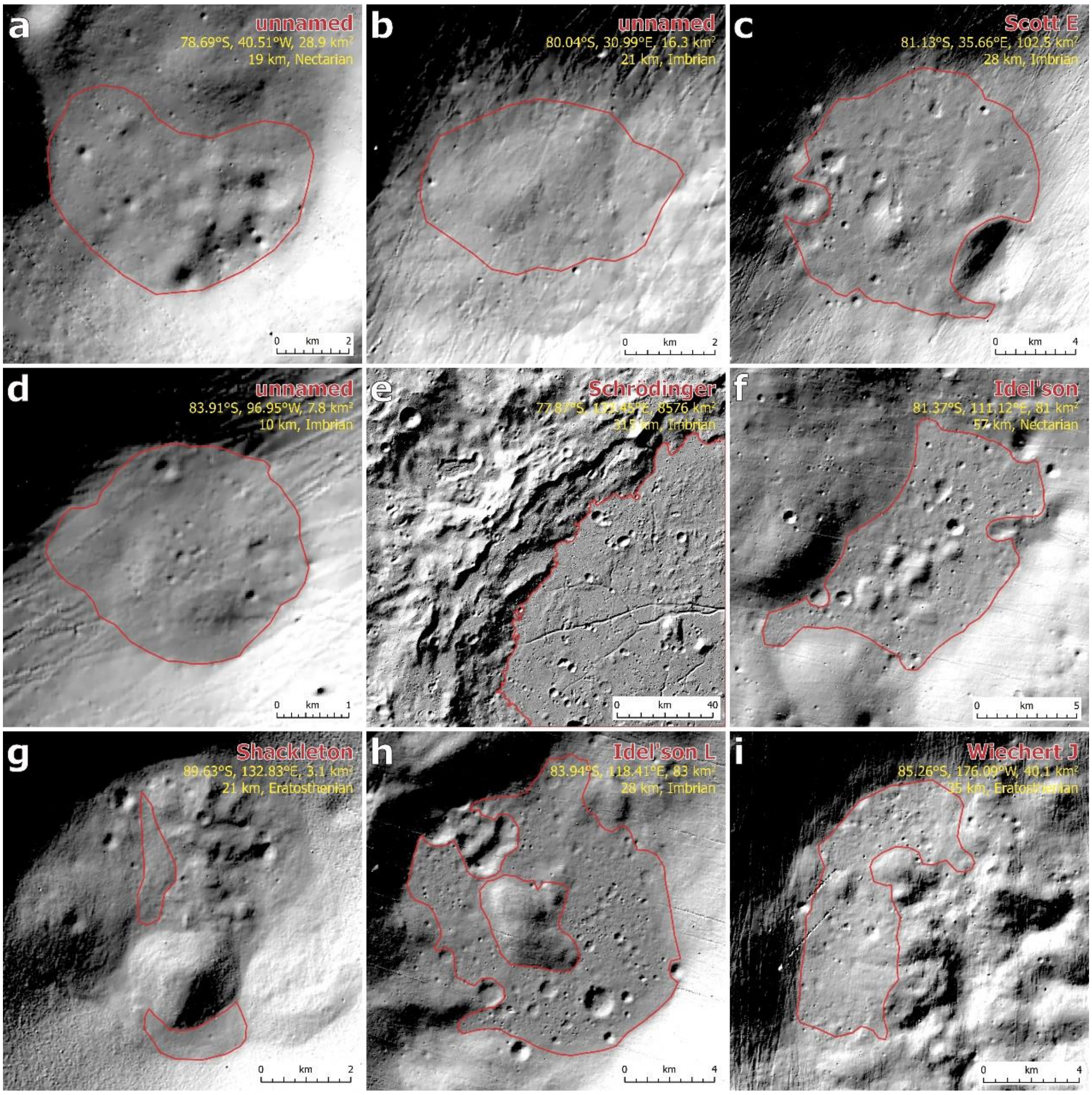

Supplementary Fig. 6. LOLA (Lunar Orbiter Laser Altimeter) shaded relief maps of the 9 sites of Schrödinger-type plain terrains (red polygons) with characteristic mound features. The center coordinate and surface area (km^2) of the plains features, and the name, diameter (km), and chronology of the host craters, are labelled in each panel.

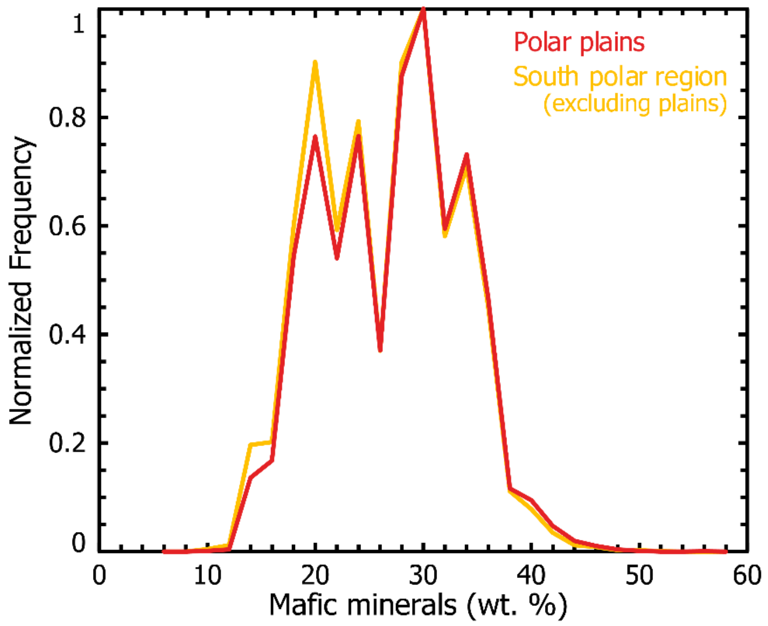

Supplementary Fig. 7. Normalized frequency of Kaguya-SP (Spectral Profiler) and LRO-LOLA (Lunar Reconnaissance Orbiter Lunar Orbiter Laser Altimeter) blended mafic mineral abundance (the sum of olivine and pyroxene abundances) for the polar plains mapped in this work and their comparison with the entire southern polar region (excluding the mapped plains).

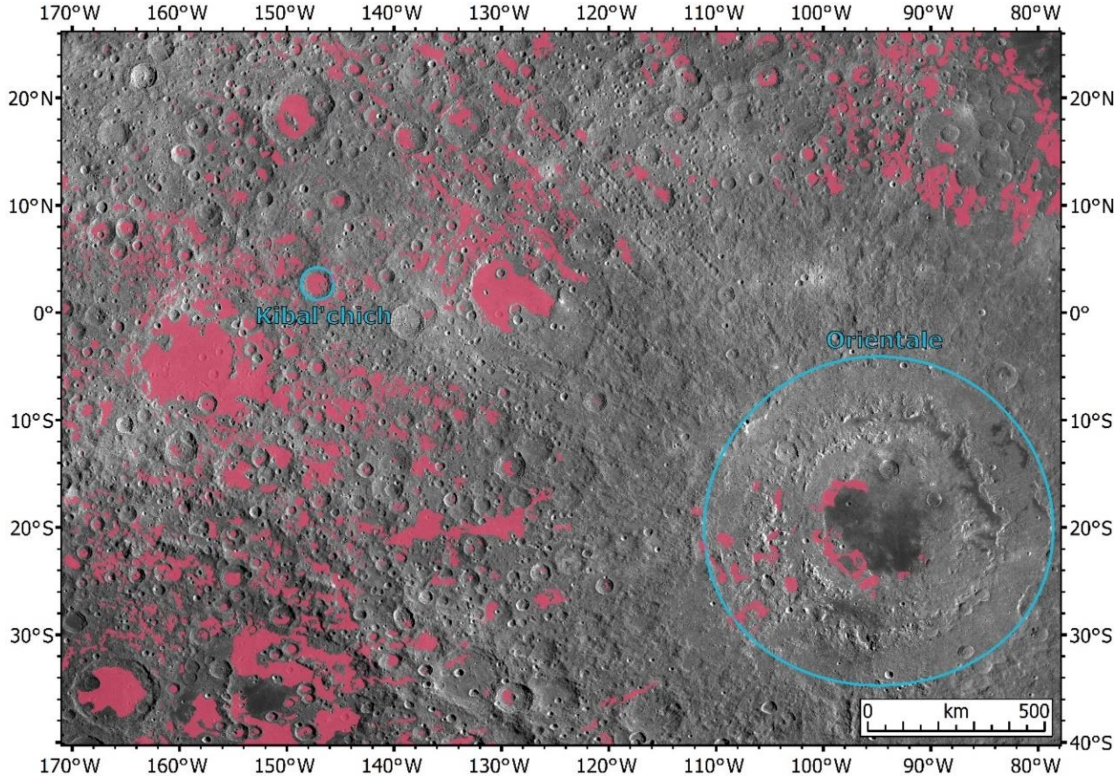

Supplementary Fig. 8. The Kibal'chich crater in the context of many light plains (pink patches)[1] radial to the Orientale basin. Basemap is a LRO (Lunar Reconnaissance Orbiter) WAC (Wide-Angle Camera) mosaic map projected into a plate carrée projection.

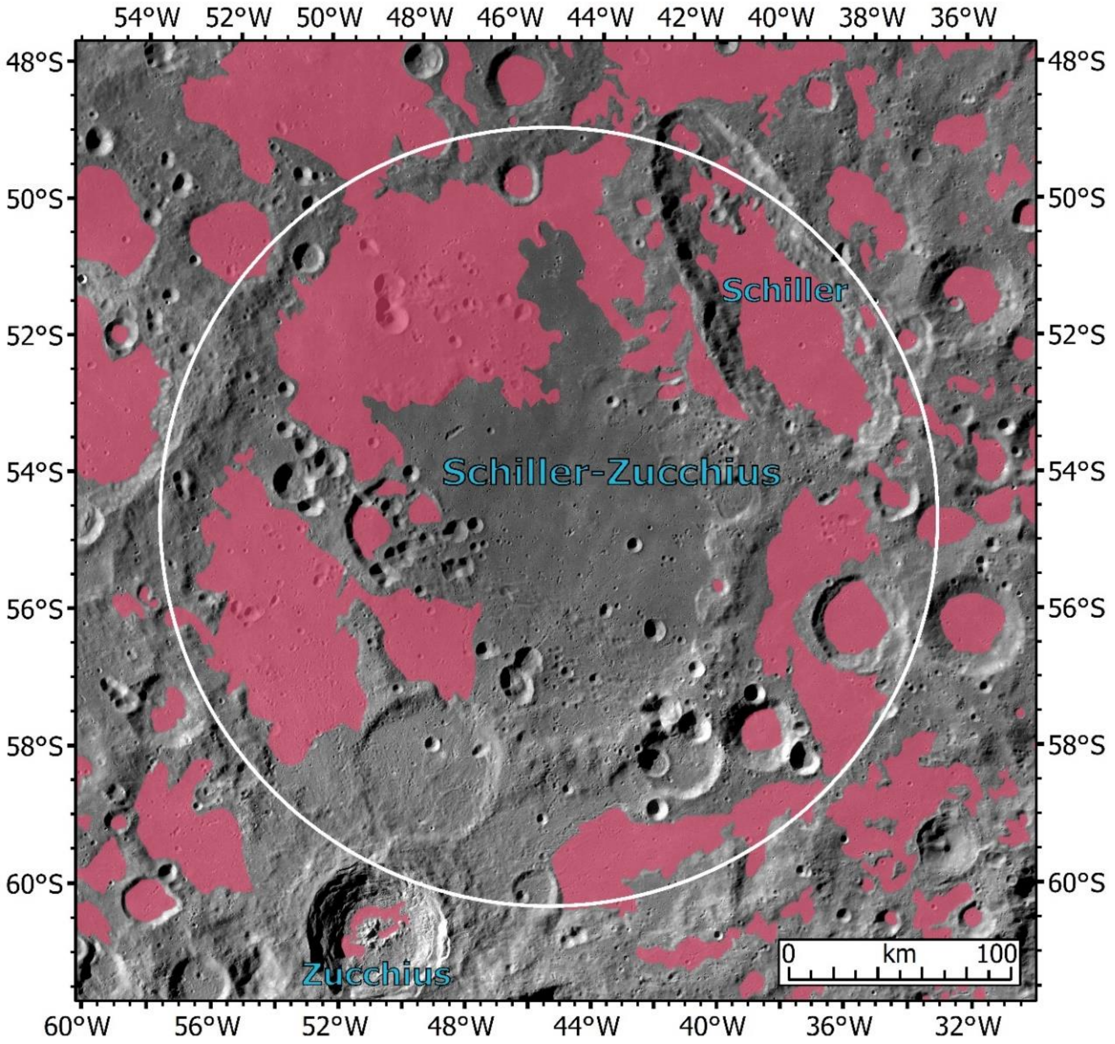

Supplementary Fig. 9. The 350-km-diameter Schiller-Zucchius (unofficial name) impact basin (centered at 55°S, 45°W) in the northeast of Zucchius crater and the mapped light plains (pink patches)[1]. Basemap is a LRO (Lunar Reconnaissance Orbiter) WAC (Wide-Angle Camera) mosaic and the projection is Lambert conformal conic, centered at the basin center.

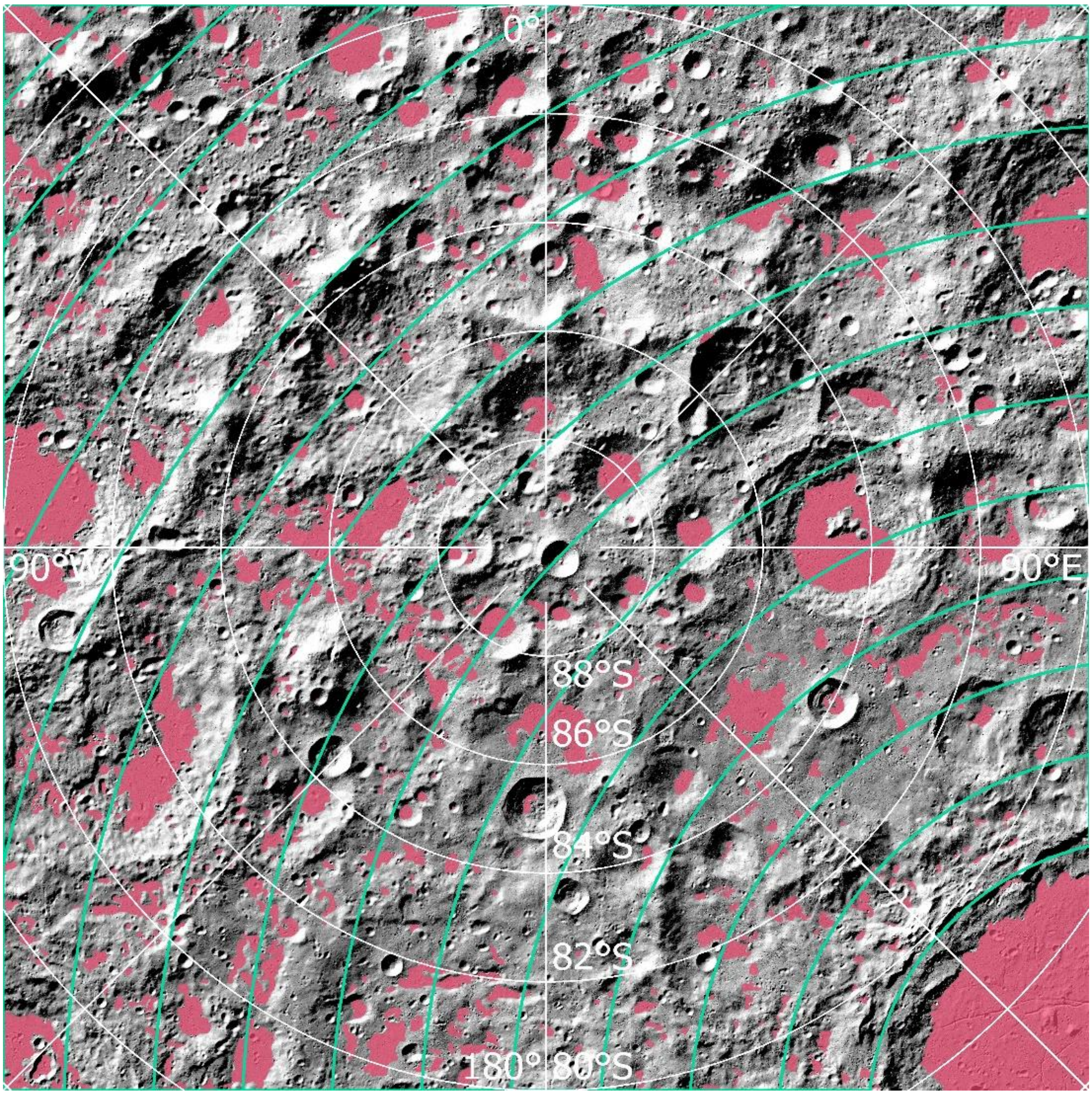

Supplementary Fig. 10. The division of the southern lunar polar region into bins (green circular arcs) with 50 km width from the Schrödinger basin rim to access the spatial distribution of the plains terrains (pink patches) with respect to the Schrödinger basin.

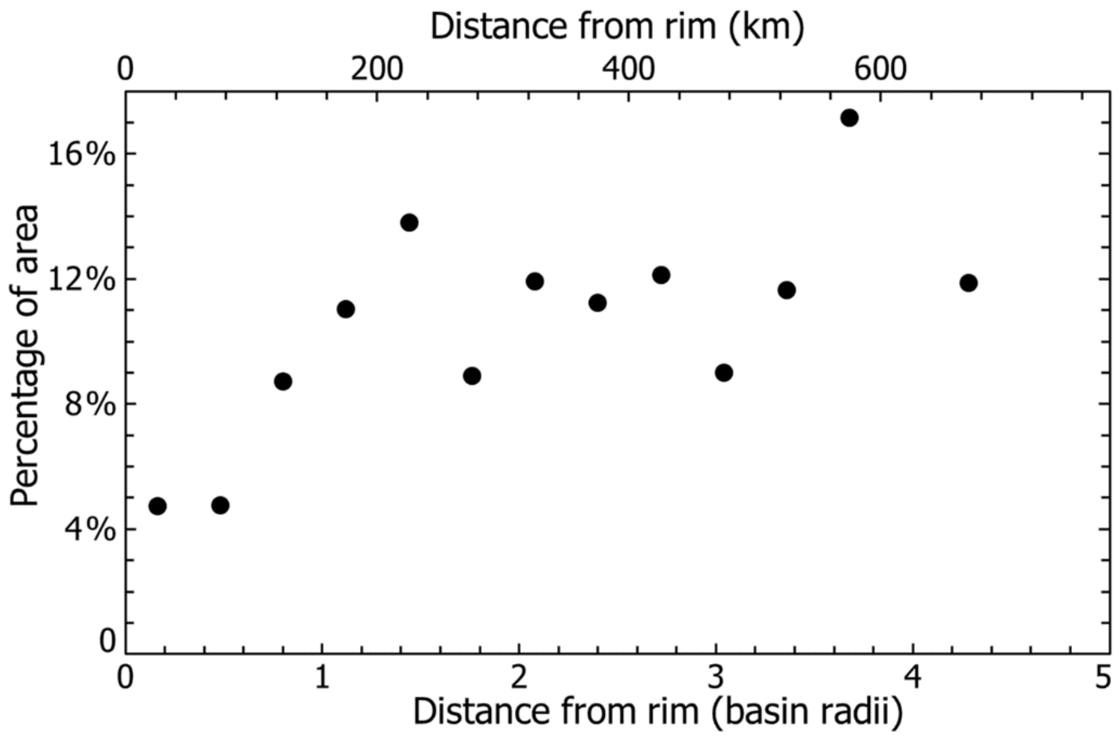

Supplementary Fig. 11. The distribution of the polar plains terrains (in percentage) relative to the distances from the Schrödinger basin rim (in basin radii and km).

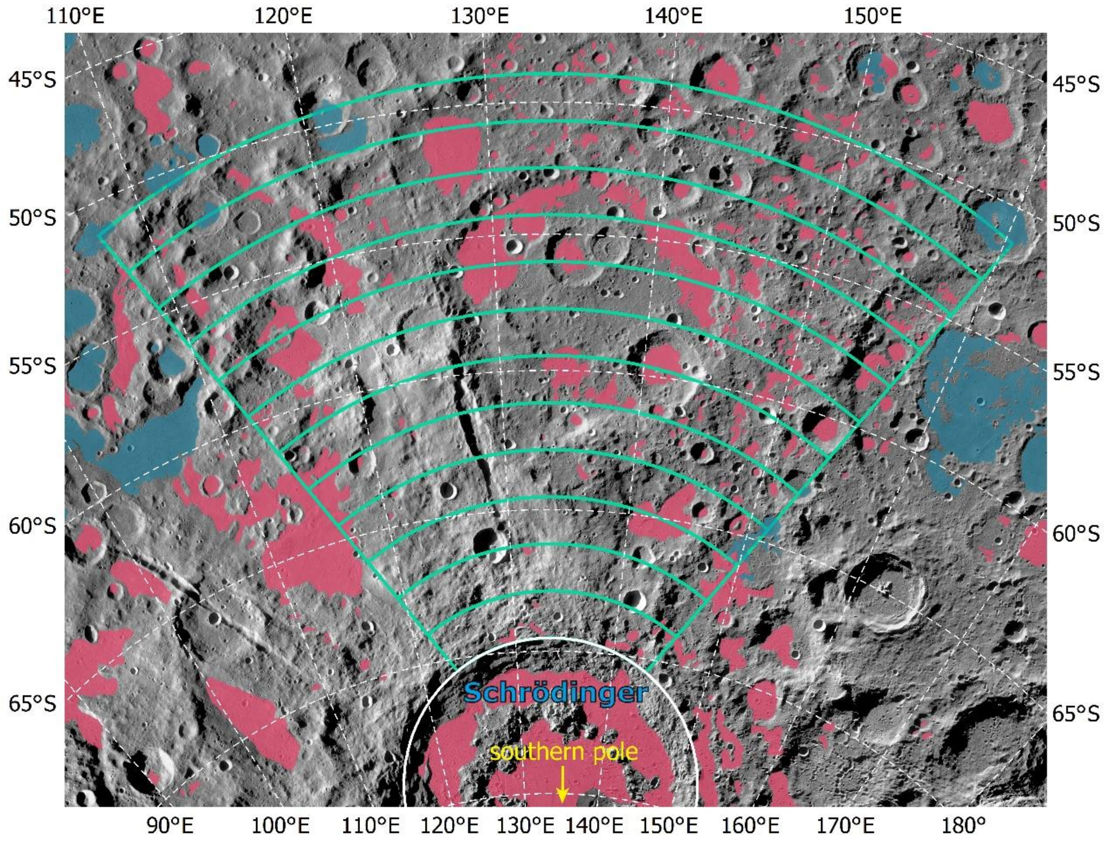

Supplementary Fig. 12. The division of the northern exterior of the Schrödinger basin into bins (green circular arcs) with 50 km width from the basin rim (white circle) to access the spatial distribution of the light plains (pink patches[1]) with respect to the

Schrödinger basin. Basemap is a LRO (Lunar Reconnaissance Orbiter) WAC (Wide-Angle Camera) mosaic and the projection is orthographic, centered at the basin center (74.6°S, 133.5°E), and mare deposits are shown as blue patches[2].

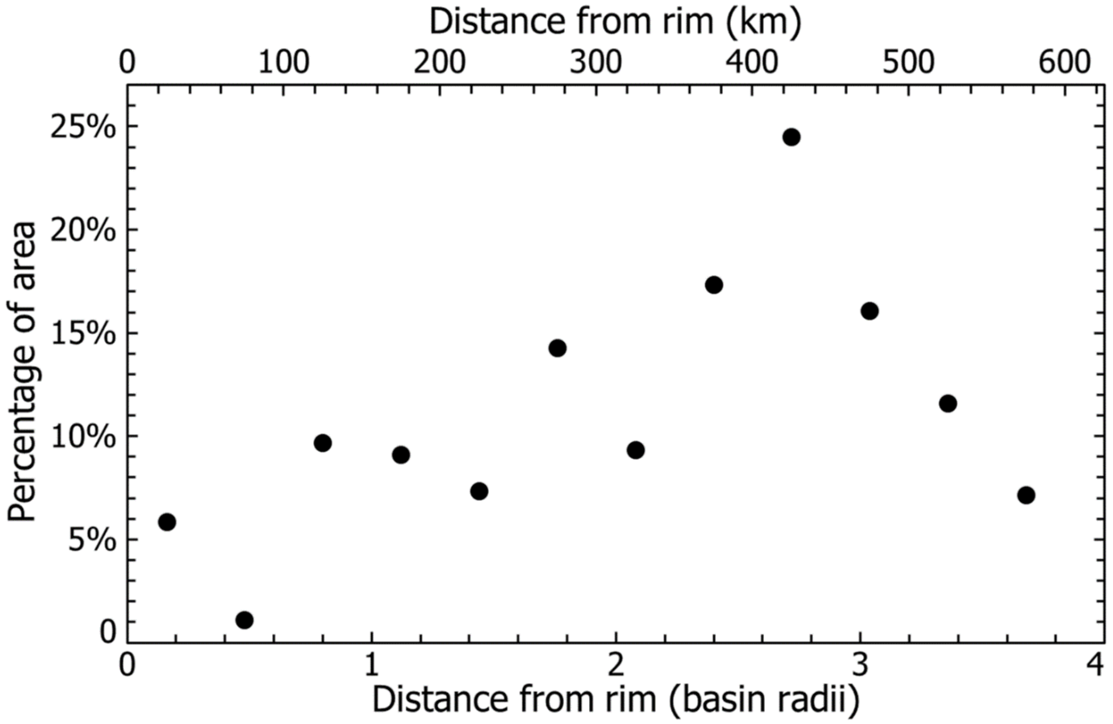

Supplementary Fig. 13. The distribution of the light plains in the northern basin exterior (in percentage) relative to the distances from the Schrödinger basin rim (in basin radii and km).

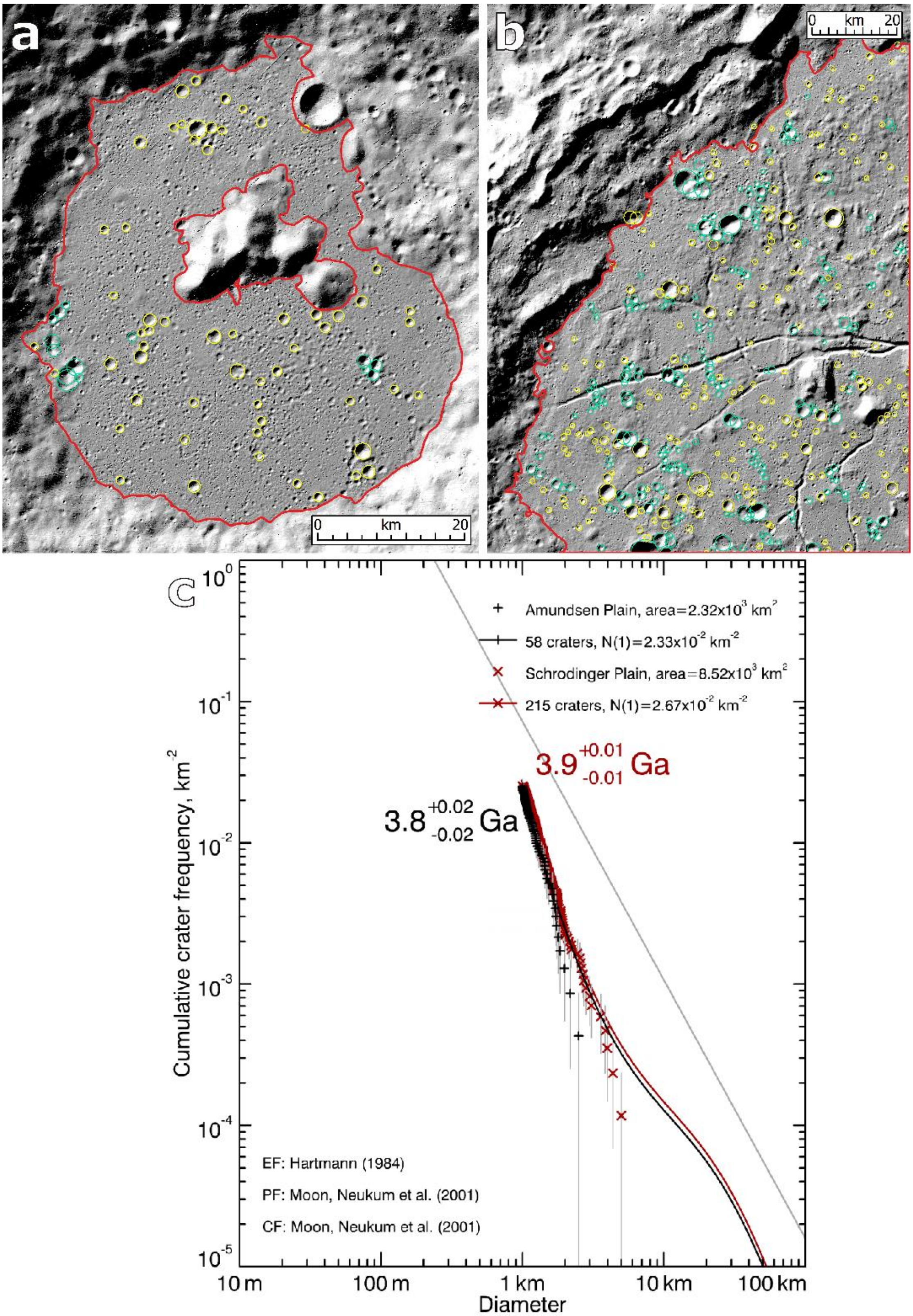

Supplementary Fig. 14. Spatial distribution of impact craters ≥1 km in diameter (from the Robbins crater catalogue[3]) superposed on the plains terrains within (a) Amundsen crater and (b) Schrödinger basin. Possible secondaries are marked with green circles. (c) Cumulative size-frequency distribution of the primary impact craters and the derived model ages. The fitting of the model age is based on the Neukum lunar PF (production function) and CF (chronology function), using the CraterStats

software package[4,5]. The gray line on the right is the lunar equilibrium function (EF) curve from Hartmann[6]. Note that the error values of the model ages are only statistical errors from the crater population measurements and do not involve the substantial uncertainties of the lunar chronology function[4,7].

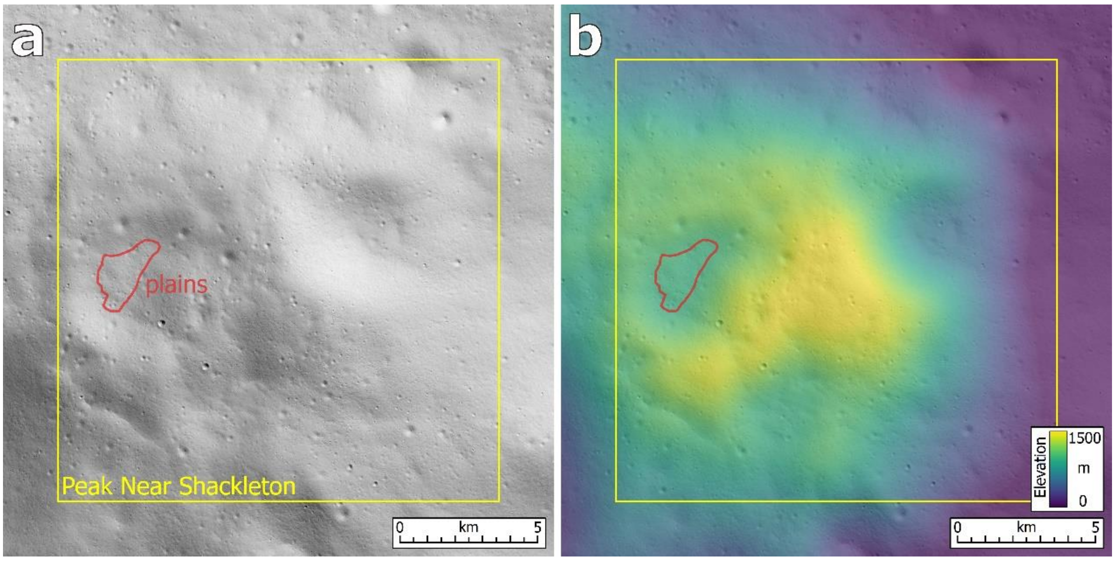

Supplementary Fig. 15. LOLA (Lunar Orbiter Laser Altimeter) (a) shaded relief and (b) topographical maps of the Peak Near Shackleton (outlined by the yellow box), one of the candidate landing region of NASA's Artemis program, which contains a small plain unit (red polygons; ~3 km^2).

Supplementary Table 1. All lunar craters or basins ≥10 km in diameter that are able to deliver ejecta materials ≥5 m in thickness to the site on the floor (85°S, 88°E) of Amundsen crater. Note that many craters/basins are stratigraphically older than the Amundsen crater and should not contribute to the plains materials on the Amundsen floor.

Crater name[1]	Stratigraphy[2]	Diameter (km)	Latitude (°)	Longitude (°)	Foreign materials thickness (m)[3]	Local materials thickness (m)[3]	Ejecta deposits thickness (m)[3]
SPA	pN	2491.9	-52.698	177.587	1407.6	9348.3	10755.9
Amundsen-Ganswindt	pN	365.9	-81.136	122.169	913.3	1306.6	2219.9
Schrödinger	I	321.5	-74.768	133.057	60.9	183.5	244.4
Hédervári	pN	73.4	-81.770	85.729	20.9	18.4	39.2
	pN	61.2	-83.602	68.171	14.4	11.8	26.2
Sikorsky-Rittenhouse	pN (?)	296.9	-69.084	111.708	13.6	53.7	67.4
Nobile	pN	77.0	-85.329	53.250	13.4	14.1	27.5
	pN	182.8	-75.326	87.103	13.4	32.8	46.2
Orientale	I	941.0	-19.830	-95.243	13.1	133.3	146.4
Crisium	N	1114.5	16.397	59.465	9.9	123.5	133.4
Mendel-Rydberg	pN (?)	654.3	-49.150	-93.653	8.9	67.2	76.1
	pN (?)	186.2	-76.156	42.874	8.2	23.7	32.0
Nectaris	N	872.0	-15.828	34.763	8.1	79.2	87.3
Smythii	pN	954.7	-1.393	86.117	8.0	87.0	95.0
Imbrium	I	1198.4	34.260	-17.144	7.5	108.9	116.4
Humorum	N	796.4	-25.958	-39.401	6.7	63.4	70.1
Scott	pN	94.7	-82.115	47.503	6.6	10.5	17.1
Demonax	N	120.1	-78.259	59.445	5.5	11.6	17.1
Tranquillitatis	pN	907.1	8.253	28.416	5.4	64.4	69.8

[1]Blanks mean unnamed craters or basins, and names in italics indicate unofficial names.

[2]The stratigraphic ages are referred to the geologic map of Fortezzo et al.[8] and Krasilnikov et al.[9], pN for Pre-Nectarian, N for Nectarian, and I for Imbrian. Question marks indicate that these craters are not resolved in the geologic map and their stratigraphy is estimated from cross-cutting relationships and topographical degradations.

[3]The foreign ejecta materials thickness of Orientale and Schrödinger basin are computed from individual measurements of Xie et al.[10] and Xu and Xie[11], and the others are computed from the ejecta distribution model of Pike[12] with distance and deposition corrections of Xu et al.[13]. All the calculations assume the symmetric distribution of ejecta thickness of each crater/basin. The thickness/depth of local materials excavated by and mixed with foreign ejecta materials are calculated using the mixing model of Oberbeck et al.[14] and Petro and Pieters[15]. The ejecta deposition thickness is the sum of the foreign and local materials thicknesses.

Supplementary References

1. Meyer, H. M., Denevi, B. W., Robinson, M. S. & Boyd, A. K. The global distribution of lunar light plains from the Lunar Reconnaissance Orbiter Camera. *J. Geophys. Res.-Planets* **125**, e2019JE006073 (2020).
2. Nelson, D. M., Koeber, S. D., Daud, K., Robinson, M. S., Watters, T. R., Banks, M. E., & Williams, N. R. Mapping lunar maria extents and lobate scarps using LROC image products. *Lunar and Planetary Science Conference* **45** 2861 (2014).
3. Robbins, S. J. A new global database of lunar impact craters >1–2 km: 1. Crater locations and sizes, comparisons with published databases, and global analysis. *J. Geophys. Res.-Planets* **124**, 871-892 (2019).
4. Michael, G. G., Kneissl, T. & Neesemann, A. Planetary surface dating from crater size-frequency distribution measurements: Poisson timing analysis. *Icarus* **277**, 279-285 (2016).
5. Michael, G. G. & Neukum, G. Planetary surface dating from crater size–frequency distribution measurements: Partial resurfacing events and statistical age uncertainty. *Earth Planet. Sci. Lett.* **294**, 223-229 (2010).
6. Hartmann, W. K. Does crater “saturation equilibrium” occur in the solar system? *Icarus* **60**, 56-74 (1984).
7. Fassett, C. I. Analysis of impact crater populations and the geochronology of planetary surfaces in the inner solar system. *J. Geophys. Res.-Planets* **121**, 1900-1926 (2016).
8. Fortezzo, C. M., Spudis, P. D., & Harrel, S. L. Release of the digital unified global geologic map of the Moon at 1:5,000,000-scale. *Lunar and Planetary Science Conference* **51**, 2760 (2020).
9. Krasilnikov, S. S., Ivanov, M. A., Head, J. W. & Krasilnikov, A. S. Geologic history of the south circumpolar region (SCR) of the Moon. *Icarus* **394**, 115422 (2023).
10. Xie, M. & Zhu, M.-H. Estimates of primary ejecta and local material for the Orientale basin: Implications for the formation and ballistic sedimentation of multi-ring basins. *Earth Planet. Sci. Lett.* **440**, 71-80 (2016).
11. Xu, L. & Xie, M. Ejecta thickness distribution of the Schrödinger basin on the Moon. *J. Geophys. Res.-Planets* **125**, e2020JE006506 (2020).
12. Pike, R. J. Ejecta from large craters on the Moon: Comments on the geometric model of McGetchin et al. *Earth Planet. Sci. Lett.* **23**, 265-271 (1974).
13. Xu, L., Zhang, X., Qiao, L. & Lai, J. Evaluating the thickness and stratigraphy of ejecta materials at the Chang'e-4 landing site. *Astron. J.* **162**, 29 (2021).
14. Oberbeck, V. R. et al. On the origin of the lunar smooth-plains. *The Moon* **12**, 19-54 (1975).
15. Petro, N. E. & Pieters, C. M. The lunar-wide effects of basin ejecta distribution on the early megaregolith. *Meteorit. Planet. Sci.* **43**, 1517-1529 (2008).
